# A Review of African Swine Fever and the Potential for Introduction into the United States and the Possibility of Subsequent Establishment in Feral Swine and Native Ticks

**DOI:** 10.3389/fvets.2018.00011

**Published:** 2018-02-06

**Authors:** Vienna R. Brown, Sarah N. Bevins

**Affiliations:** ^1^Oak Ridge Institute for Science and Education (ORISE), Oak Ridge, TN, United States; ^2^Wildlife Services, National Wildlife Research Center (NWRC), Animal and Plant Health Inspection Service, United States Department of Agriculture (USDA), Fort Collins, CO, United States

**Keywords:** African swine fever, viral introduction, emergency preparedness, surveillance, domestic pigs, feral swine

## Abstract

African swine fever (ASF) is caused by African swine fever virus (ASFV), which can cause substantial morbidity and mortality events in swine. The virus can be transmitted *via* direct and indirect contacts with infected swine, their products, or competent vector species, especially *Ornithodoros* ticks. Africa and much of Eastern Europe are endemic for ASF; a viral introduction to countries that are currently ASF free could have severe economic consequences due to the loss of production from infected animals and the trade restrictions that would likely be imposed as a result of an outbreak. We identified vulnerabilities that could lead to ASFV introduction or persistence in the United States or other ASF-free regions. Both legal and illegal movements of live animals, as well as the importation of animal products, byproducts, and animal feed, pose a risk of virus introduction. Each route is described, and current regulations designed to prevent ASFV and other pathogens from entering the United States are outlined. Furthermore, existing ASFV research gaps are highlighted. Laboratory experiments to evaluate multiple species of *Ornithodoros* ticks that have yet to be characterized would be useful to understand vector competence, host preferences, and distribution of competent soft tick vectors in relation to high pig production areas as well as regions with high feral swine (wild boar or similar) densities. Knowledge relative to antigenic viral proteins that contribute to host response and determination of immune mechanisms that lead to protection are foundational in the quest for a vaccine. Finally, sampling of illegally imported and confiscated wild suid products for ASFV could shed light on the types of products being imported and provide a more informed perspective relative to the risk of ASFV importation.

## Key Points

- African swine fever (ASF) is caused by African swine fever virus (ASFV), which is the only known arthropod-borne DNA virus.
- Currently, ASF is not present in the United States, but it is a high consequence, foreign, notifiable swine disease, and the economic consequences associated with an introduction could be catastrophic.
- The virus is endemic in many parts of the world, including most of sub-Saharan Africa, the island of Sardinia, and parts of the Caucasus region and Eastern Europe.
- The routes of concern for the introduction of ASF into the United States are the legal or illegal importation of live animals (or their products) or a bioterrorism event.
- Introduction or spillover events from domestic swine into feral swine populations would substantially complicate the eradication process as would infection in native *Ornithodoros* tick species.
- Currently, there is no ASF vaccine approved for use.
- Future research should involve (1) laboratory feeding experiments to evaluate multiple species of North American *Ornithodoros* ticks that have yet to be characterized, (2) expanded analyses to explore the distribution and host preferences of competent soft tick vectors in relation to high pig production regions as well as high densities of feral swine, (3) characterization of antigenic viral proteins that contribute to a host immune response and determination of immune mechanisms that lead to protection, (4) expanding classical swine fever slaughter surveillance and random blood collections to include screening for ASFV, in the event of an increased risk of viral introduction, and (5) sampling of wild suid products that were illegally imported and confiscated for the detection of ASFV.

## Introduction

African swine fever (ASF), first described in Africa in the 1920s, is caused by African swine fever virus (ASFV). Infection results in high morbidity and mortality in swine and has drastic implications for global domestic swine production (1). This disease is reportable to the World Organisation for Animal Health (OIE), and viral infection in swine can have severe economic consequences associated with production losses, trade limitations, and eradication programs (2). Currently, the United States is ASFV free. This article outlines what is known about ASFV and aims to describe existing gaps in knowledge. Finally, a summary of US vulnerabilities for viral introduction and persistence is provided. Countries with endemic ASF in domestic swine likely have a different set of challenges compared to the United States and other ASF-free regions and may benefit from the development of disease control methods that are commonly used, such as enforceable quarantine zones, diagnostic assays, and culling of infected animals. However, eradicating a disease that is established in a wild population, such as wild boar, is highly complex and depends on a deep understanding of the disease ecology within a specific epidemiological context.

## Virus Description

African swine fever virus is a large, enveloped virus in the Asfarviridae family that causes hemorrhagic diseases in domestic pigs and several species of wild swine (3). The virus is a genetically complex double-stranded DNA virus that contains a series of genes used for virulence, immune evasion, and cell process modulation (4). Twenty-three genotypes have been described based on the partial sequences of the p72 gene (5, 6). All 23 genotypes are present in Africa, whereas only genotypes I and II have been found outside of that continent. The virus primarily infects cells of the mononuclear phagocytic system (monocytes and macrophages) and replicates in the cytoplasm. The endoplasmic reticulum is believed to play an important role in viral assembly and ASFV envelopment (3, 4, 7).

## Transmission and Clinical Disease

African swine fever virus can be transmitted *via* direct contact with infected animals, either domestic swine or wild boar, indirect contact *via* contaminated fomites or uncooked meat from infected animals, or through arthropod vectors, particularly soft tick species in the genus *Ornithodoros* (1, 8). The virus is highly stable in proteinaceous environments and quite resistant to high temperatures, requiring 60°C for 20 min for inactivation. Domestic pig-to-pig transmission is thought to occur primarily through infection of the upper respiratory tract as domestic pigs have been shown to shed infectious virus from all secretions and excretions, with particularly high concentrations in the oronasal fluid. ASFV is very persistent in blood and tissues after death; thus, an opportune vehicle to transmit infection is feeding uncooked swill. Environmental contamination following necropsies, pig fights that result in bloodshed, or bloody diarrhea following infection may also serve as a route for new infections. Airborne transmission has been demonstrated in a laboratory setting where animals were densely housed (9).

*Ornithodoros* ticks have also been found to serve as biological vectors for ASFV, with documented transstadial, transovarial, and sexual transmission (10). In some regions of Africa, ASFV cycles between juvenile common warthogs and *Ornithodoros porcinus porcinus* ticks, which inhabit their burrows. In Europe, *Ornithodoros erraticus* have been found to vector ASFV and were involved in the disease epidemiology on the Iberian Peninsula between the 1960s and 1990s; however, *O. erraticus* are not involved in the current ASF scenario in Eastern Europe and Sardinia. Biting flies, particularly *Stomoxys spp*, have been found to be capable of mechanical transmission for ASFV (11).

Domestic swine, Eurasian wild boar, warthogs, bushpigs, and giant forest hogs are all susceptible to infection with ASFV; however, warthogs and bushpigs generally develop asymptomatic infections and serve as a viral reservoir, in what is often referred to as the sylvatic cycle (12). Peccaries are thought to be resistant to infection. Neonatal warthogs develop a sufficient viremia to infect new ticks but do not develop clinical disease, and adult warthogs are impervious to the pathogenic effects of the virus although the virus can be often extracted from their lymph nodes (13). ASFV has a predilection for lymph nodes near the head, and warthogs remain infected for life (14). Neither horizontal nor vertical transmission has been documented in warthogs, with soft ticks serving as the sole route of transmission between infected and susceptible warthogs (1, 15, 16). Sexual transmission is not indicated in warthogs; however, the virus is found in genital secretions and so it remains a possibility (1). To date, there has been no conclusive data suggesting a long-term carrier state; however, a survey conducted in central Kenya found ASFV [detected *via* polymerase chain reaction (PCR)] in asymptomatic domestic swine and warthogs (17).

Experimental infection of bushpigs demonstrated the absence of clinical disease despite a robust viremia lasting 35–91 days following infection with ASFV, which was sufficient to infect *O. porcinus porcinus* ticks that fed on the bushpigs during their viremic period (18). Infected ticks were able to transmit ASFV to naive domestic pigs. Certain strains of ASFV in experimentally inoculated bushpigs were capable of transmission *via* direct contact with domestic swine, whereas other strains were not. Infected domestic swine were not able to transmit the infection to in-contact bushpigs, suggesting that they are not as readily infected *via* direct contact compared to domestic swine.

Clinical disease can manifest in multiple ways ranging from death with no signs (peracute, mortality ~100%) to an asymptomatic infection; however, most isolates of ASFV cause acute hemorrhagic fever in domestic pigs and result in mortality nearing 100% (1, 19). All age groups of pigs have been found to be equally susceptible to ASFV infection, as opposed to classical swine fever virus (CSFV) where young pigs are much more susceptible (20). Acute infections are caused by highly virulent strains and are typically characterized by a high fever, anorexia, lethargy, weakness, recumbancy, diarrhea and/or constipation, abdominal pain, hemorrhagic signs, respiratory distress, nasal and conjunctival discharge, and abortions in pregnant females. Death often occurs within 7–10 days after the onset of clinical signs. Depending on the virulence of the ASFV strain, acute infections are often the predominant form at the beginning of an outbreak in disease-free regions; however, once established, the disease often progresses to subacute clinical forms that can be sustained over time (20). It is important to note that this pattern has been previously observed although it is not the established truth. Moderately virulent strains result in subacute infection (often with high mortality in young animals and much lower mortality in older animals) where the clinical signs often include abortion, fever, and transient hemorrhaging with death or recovery occurring within 3–4 weeks. Chronic infections (mortality is very low) are characterized by intermittent or low fever, appetite loss, and depression and, in some instances, result in a fatal infection. Animals that remain persistently infected for months, such as survivors or subclinically or chronically infected pigs, may play a role in disease persistence in endemic regions. Also, it has been speculated that they may contribute to sporadic outbreaks and introductions to ASFV-free zones (20).

Domestic pigs are most infectious during the incubation period and may shed virus for >48 hours prior to the presentation of clinical disease (1). Recovered pigs may shed infectious virus for 1 month after the disappearance of clinical signs. Pig populations that have developed a degree of resistance to the virus are better able to maintain and circulate ASFV as the disease is not self-limiting (21, 22).

## Global Distribution and Epidemiology

African swine fever was restricted to the African continent from its first description in the 1920s until 1957 when an outbreak was reported in Portugal (23). This outbreak was effectively controlled and eradicated until a second recurrence in 1960, which resulted in ASF being endemic in the Iberian Peninsula (Portugal and Spain) until 1995. During the 1970s and 1980s, ASF emerged in several parts of the world, including other European countries (the Netherlands, Italy, France, and Belgium) and the Americas (Cuba, the Dominican Republic, Haiti, and Brazil) (16). This global spread is thought to be due largely to feeding domestic animals contaminated pork products that entered each region *via* international air and seaports. After establishment in domestic swine herds, infected pigs and pork products became the primary source of infection.

On the basis of the ability of ASFV to be transmitted *via* direct and indirect contacts and through an arthropod vector, Sánchez-Vizcaíno et al. (23) outline five epidemiological scenarios and examples of regions where each type of situation occurred, depending on the existence of wild reservoirs and competent tick vectors. The first scenario involves the original natural cycle and describes transmission in Eastern and Southern Africa in which a sylvatic cycle occurs between wild suids, especially warthogs, and *O. porcinus porcinus* ticks. Spillover into domestic swine is typically associated with infected tick bites or ingestion of contaminated warthog meat. A second scenario describes transmission occurring primarily through direct contact between infected and susceptible domestic pigs and indirect contact between susceptible pigs and contaminated pork products. Ticks are not involved. This describes ASF dynamics in many West African countries. Third, as was observed on the Iberian Peninsula, both wild boar and domestic pigs were infected, and transmission primarily occurred *via* direct contact between infected and susceptible animals and *via* the consumption of infected meat. *O. erraticus* contributed to transmission in outdoor production systems; however, this tick species is only capable of transstadial transmission but not transovarial, and therefore, their vector competency is lower than *O. porcinus porcinus*. Between 1968 and 1980 in Central and South America, a fourth scenario was observed in which the disease only affected domestic pigs and neither wild suids nor ticks were involved. This scenario is much easier to eradicate compared to all others. The fifth scenario occurred in Russia and the trans-Caucasian countries where both wild boar and domestic pigs were involved in transmission but ticks were not found to be involved. Most outbreaks were found in domestic pigs and were linked to movements of affected animals and their products. Understanding the epidemiology of disease, specific to the region of interest, is crucial as the development of emergency control and eradication plans are dependent upon disease transmission patterns and risk factors.

## Immune Response to ASFV

Infection with ASFV is characterized by severe immunosuppression and apoptosis, primarily replicating in monocytes and macrophages, and is believed to enter cells *via* receptor-mediated endocytosis (24, 25). Activated macrophages release IL-1, IL-6, and TNFα, which all contribute to acute-phase reactions, inflammation, activation of endothelial cells, and apoptosis (26). Similar cell tropism and organ distribution have been observed across all strains of ASFV; however, more severe tissue destruction is associated with strains of increasing virulence. Neutralizing antibodies and CD8^+^ T cells and natural killer cells are believed to play an important role in the host immune response against ASFV. *In vitro* experiments suggest that some cellular mechanisms are regulated by ASFV *via* the encoding of specific regulatory genes and by interaction with viral and cellular proteins; however, most cellular functions altered after infection remain unknown (25). Proteomic evaluation demonstrated that ASFV shuts down the majority of protein synthesis, affecting approximately 65% of cellular proteins. Specific cellular proteins were found to be overexpressed after ASFV infection, and most were involved in redox homeostasis, programmed cell death, and coagulation.

The role of neutralizing antibodies has been evaluated, and results are variable. Passive transfer experiments performed in domestic swine by Onisk et al. (27) found that 85% of pigs that received the anti-ASFV IgG survived challenge compared to 0% of unimmunized controls. Treated animals underwent transient fever but otherwise appeared clinically normal. Viremia in pigs that received the antibody transfer was found to be delayed and reduced.

Viral neutralizing epitopes were identified on three viral capsid proteins—p30, p54, and p72—and domestic swine were immunized using a baculovirus expressing each of these proteins prior to challenge with a homologous virus (28). Immunized animals were found to have a 2-day delay in the onset of clinical disease and a reduced viremia, but there was no effect on disease development, progression, or outcome. The authors concluded that neutralizing antibodies to these ASFV proteins are insufficient for antibody-mediated protection.

The findings by Onisk et al. (27) and Neilan et al. (28) appear to be in stark contrast to one another, and differences are believed to be due in part to variations in virus strains (and subsequently, virulence) and challenge doses. The relative role of neutralizing antibodies may be dependent on the virulence of the ASFV isolate used, with neutralizing antibodies providing a more protective response against less virulent strains. However, large differences in study design between the two experiments make comparison very difficult as Onisk and colleagues used passive transfer, which is a mixture of numerous antibodies compared to Neilan et al. (28) who immunized swine with specific epitopes. Much further characterization of the role of antibodies is required.

Interestingly, in northern Mozambique, a region endemic for ASF, a population of domestic pigs were found to have high levels of circulating antibodies to ASFV (29). A group of pigs from this population were collected and their offspring were evaluated through experimental ASFV challenge for the heritability of this resistance to ASF. The offspring were acutely susceptible to challenge with a virulent strain of ASFV, suggesting that the ASFV resistance in the parental population was not heritable. The authors hypothesize that this observed resistance is resultant from (1) prior exposure to a less virulent but antigenically similar field virus prior to exposure to a virulent strain, (2) maternal antibody resistance, (3) exposure to small quantities of infectivity that may result in a sublethal infection that confers immunity to a subsequent challenge (29).

## Vector Biology

As stated previously, several soft tick species have been implicated in ASFV transmission in endemic and outbreak regions. It is important to note that the taxonomy of the *O. porcinus porcinus* ticks has changed over time on the basis of both morphological and biological characteristics. Prior to 1979, *O. porcinus porcinus* ticks were often referred to as *Ornithodoros moubata porcinus* or simply *Ornithodoros moubata*. The *O. moubata* complex was then split into four distinct species, including *Ornithodoros porcinus*, which was further divided into *O. porcinus porcinus* and *Ornithodoros porcinus domesticus* (30). However, in much of the current literature *O. moubata* and *O. porcinus porcinus* are used interchangeably.

Plowright et al. (31) demonstrated that *O. porcinus porcinus* could be infected with multiple strains of ASFV and develop a persistent infection although the minimum infective dose varied between strains. Furthermore, experimental challenges confirmed that infected ticks could readily transmit ASFV to domestic pigs. Later studies determined that *O. porcinus porcinus* could transmit the infection transovarially; however, there was tremendous variability between egg batches from different ticks and between successive egg batches from the same tick (32). Interestingly, it was found that the prevalence of infected eggs increases after each successive infected blood meal. *O. porcinus porcinus* ticks have been found to maintain high ASFV titers over time, and no cytopathological lesions have been observed in these ticks, suggesting that *O. porcinus porcinus* ticks and ASFV are co-adapted and likely represent a co-evolved system (33). ASFV follows a common virus–tick pathway upon ingestion of an infective blood meal, viral replication in the midgut, escape into the hemocoel, and infection of the coxal and salivary glands (34).

While *O. porcinus porcinus* ticks are involved in the sylvatic cycle of ASFV with warthogs, other *Ornithodoros* species are capable of transmitting infection. *O. erraticus*, found in the Mediterranean and Middle East, was implicated in ASFV transmission, and longitudinal monitoring found higher titers over time, which is suggestive of viral replication (35).

Several *Ornithodoros* species are indigenous to North and Central America, as well as the Caribbean. Experimental infections in *Ornithodoros coriaceus, Ornithodoros parkeri*, and *Ornithodoros turicata* (Americas) and *Ornithodoros puertoricensis* (Caribbean) have been performed with multiple ASFV isolates (33). *O. coriaceus* ticks were infected with five different isolates of ASFV, and viral persistence was found to range between 77 and 463 days, with transmission to domestic swine demonstrated at 502 days postinfection with the DR II strain. *O. parkeri* were challenged with one strain of ASFV and found to be infected for 46 days postinfection, whereas *O. turicata* were found to be infected for 23 days postinfection. *O. puertoricensis* ticks were infected with a single isolate of ASFV and demonstrated transmission to domestic pigs at 239 days postinfection. In addition, transovarial and transstadial transmission were demonstrated; however, transmission rates decreased with each molt. Importantly, despite the presence of *O. puertoricensis* in Haiti and the Dominican Republic, it did not appear to complicate ASFV eradication in 1978, likely due to the lack of contact between infected pigs and ticks (36). For a comprehensive overview of vector competency with different isolates of ASFV, please see the study by Kleiboeker and Scoles (33).

## ASF and European Spread

African swine fever is endemic in much of Africa but was first introduced outside of the African continent into Portugal in 1957 and again in 1960 (37). The most likely route of introduction was *via* ASFV-contaminated swill as this is a very effective means of spreading the virus over long distances. The disease was first discovered in swill-fed swine near the Lisbon airport, which furthers the hypothesis that the virus was introduced *via* this route. ASFV then spread to Spain and remained endemic on the Iberian Peninsula until the 1990s. Once introduced, ASF is especially difficult to eradicate due to the presence of wildlife reservoirs and competent soft tick vectors, the lack of a vaccine, and insufficient laboratory support for rapid and accurate diagnosis (38). It is important to note that the role of wild boar in the maintenance and transmission of ASFV varies significantly based on disease epidemiology and ecology. Wild boars were involved to some extent in the epidemiology of ASF on the Iberian Peninsula, but they did not appear to complicate control measures, which is in strict contrast to the current scenario in Eastern Europe where ASF has become established in wild boar populations independent of domestic pigs (39). Between 1960 and 1986, the disease emerged in a variety of European countries, including France, Madeira, Italy, including the island of Sardinia, Belgium, the Netherlands, and Malta (37, 40, 41). Extensive control has led to eradication in these countries, except for Sardinia, where the disease has been endemic since 1978 (20).

In June 2007, ASFV was introduced to the Caucasus region of Georgia, presumably from catering waste containing infected meat from ships docked at the Black Sea Port of Poti (38). The virus spread quickly throughout the country and by July 2007, ASFV was found in 56 of the 61 districts in Georgia. By August 2007, ASF was found in neighboring Armenia and by November 2007 was found in Azerbaijan and Russia. In 2014, outbreaks were reported in parts of the European Union, including Poland, Lithuania, Latvia, and Estonia and the first detections in each of these countries were in wild boar found dead (42). Epidemiological investigations from Lithuania and Latvia suggest that fresh grass and seeds contaminated with ASFV from infectious wild boar served as the source of infection for pigs on backyard farms (43). The viral amplification in backyard pigs then served as a viral source for other backyard farms and commercial piggeries. In 2017, ASF was reported in the Czech Republic and Romania, and between January and September 2017, the Animal Disease Notification System received notifications of about 3,700 cases in wild boar and approximately 140 cases in domestic swine from the 6 EU member states, including Sardinia (44). Interestingly, models of the most current epidemiological situation suggest that the most important risk estimator for ASF spread into disease-free EU countries is wild boar habitat and the least significant estimator is wild boar density; thus, indicating that the presence of wild boar is more important than density (45). This model can be used to identify countries that are at higher risk for ASF introduction through wild boar.

Experimental inoculation of wild boar with an ASFV isolate from the Caucasus region found that the infection resulted in uniform lethality, and the authors concluded that this highly virulent strain would be unsuitable for viral endemicity within the native population of wild boar (46). Despite this assertion, field observations show that the virus can persist independently in wild boar despite high virulence. Importantly, a low-dose challenge of wild boar with Caucasus region isolates of ASFV were found to be sufficient to result in infection of weak or runted animals (47). Once infected, these poor-doing wild boar could then serve to amplify ASFV to levels that were capable of infecting apparently healthy herd mates. The exact mechanism with which highly virulent strains of ASFV are being maintained in wild boar populations is unknown; however, in several epidemiological scenarios, it has become clear that ASFV can persist independently in wild boar populations.

Competent vector species, namely *O. erraticus* ticks, found on the Iberian Peninsula also contribute to difficulty eradicating the virus once introduced. Portugal was declared free of ASFV in 1993, but the virus re-emerged on a single farm in 1999 and ASFV-infected *O. erraticus* ticks in 1993 are suggested as the route of introduction (48). Ticks were collected from farms that were depopulated due to ASF and evaluated for their capacity to maintain an ASFV infection and transmit to susceptible domestic swine. Cell culture was used to evaluate tick infection, and four adult ticks were found to be positive using cell culture alone and another six adult ticks were found to be positive using both cell culture and PCR or direct immunofluorescence. 8.8% of tested farms were found to have infected ticks, and this infection could lead to virus isolation 2.5–5.25 years following the last possible ASFV exposure. Transmission to susceptible domestic swine occurred 2.3 years after the last possible exposure to ASFV. These findings suggest that the current European Administration regulations on ASF, where an infected property can be restocked 40 days after an outbreak in the absence of soft tick vectors, and the requirement of a 6-year quarantine if soft tick vectors are present, are appropriate (49). Furthermore, it is a distinct possibility that long-lived ASFV-infected *O. erraticus* ticks caused the single farm outbreak of ASF in 1999 after the eradication in Portugal. However, it is important to note that this finding was related to a very old and traditional housing of pigs, using pig sties in which soft ticks could become established. By using modern pig production methods, a soft tick infestation is unlikely.

## Domestic Swine in the United States

**Figure 1:**
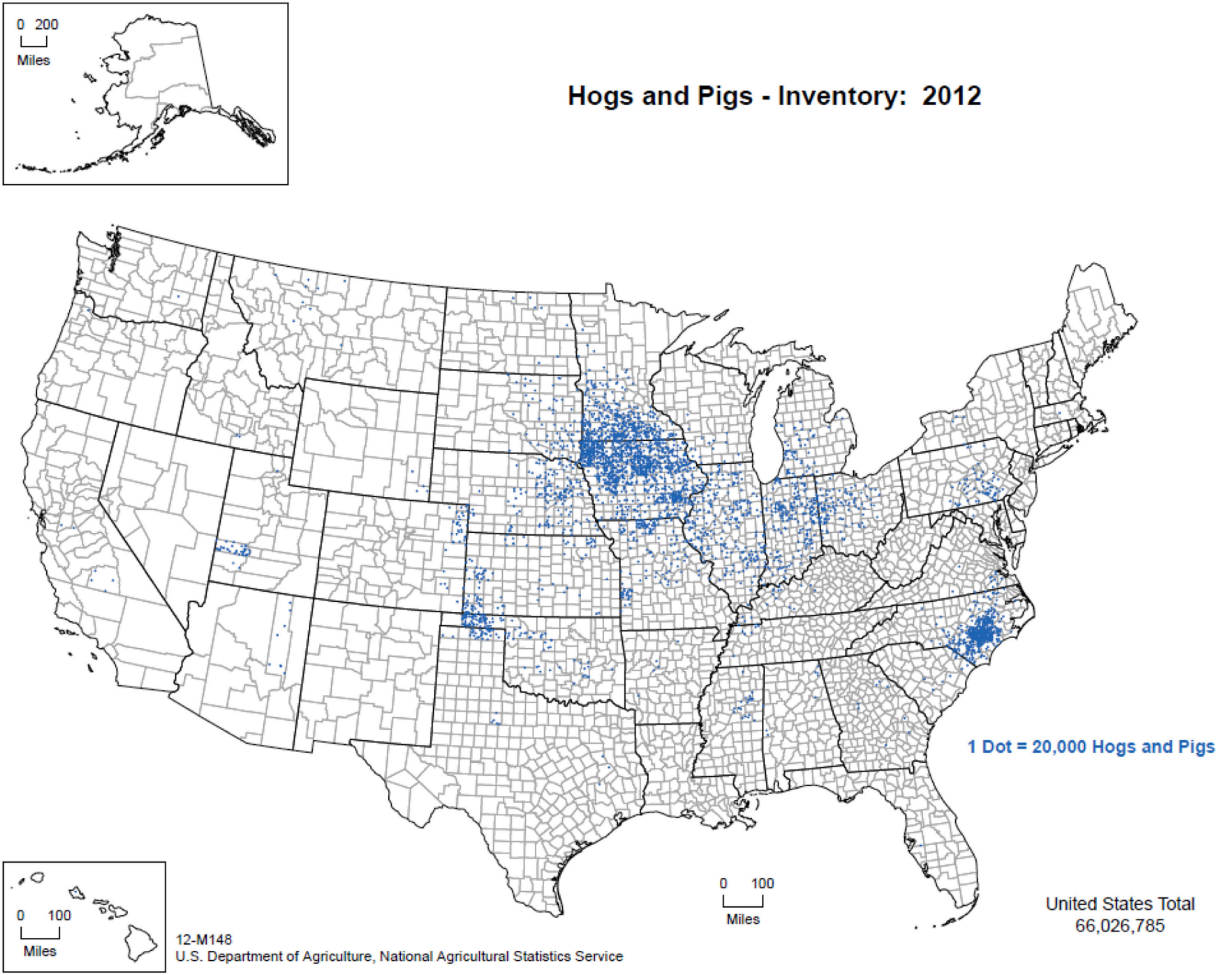
Distribution of pig production within the United States, 2012. (Figure courtesy by United States Department of Agriculture, National Agricultural Statistics Service (2015), used with permission.)

The overwhelming majority of the 65 million pigs in the United States are managed indoors under high biosecurity conditions. Figure 1 illustrates the distribution of pig production within the United States from 2012 (50), with Iowa, North Carolina, Minnesota, Illinois, and Indiana being the five top pork producing states annually. Commercial swine production is a closed system from farrowing through slaughter as a means to reduce the risk of pathogen introduction (Personal communication, 2016). Animal feed, transport vehicles, personnel, and other fomites are also closely managed to limit cross-contamination. However, it is important to note that despite the biosecurity measures in place in the commercial swine industry, porcine epidemic diarrhea virus (PEDV) entered the United States in 2013, and epidemiological analyses suggest that transport equipment contributed to viral spread (51). Moreover, in summer 2014 in northeast Lithuania, ASFV was introduced to an industrial pig farm that was intensively managed with a closed cycle and very strict biosecurity measures resulting in the death or euthanasia of >20,000 pigs (20). These examples demonstrate that despite stringent biosecurity protocols and a vertically integrated industry, it can still be difficult to control pathogens.

For ASF vector-borne transmission, however, it is unlikely that ticks would interact with domestic swine raised in commercial facilities. However, hobbyists and backyard farmers often have domestic swine that are not managed with intensive biosecurity and thus are likely to be exposed to environmental elements and other domestic livestock, wildlife species, or their feral counterparts (52). Exposure to potential soft tick vectors and other blood feeding arthropods is plausible depending on both the geographical region and the management practice. Given these conditions, an ASF introduction into the United States may put backyard farms more at risk compared to commercial facilities, as has been reported in much of Eastern Europe and some European Union member states (e.g., Latvia) (43).

## Diagnostics

Virus isolation can be used for the diagnosis of ASF as live virus can be obtained from live animals or necropsy tissues although this method is typically only used in reference laboratories to confirm diagnosis (53). The spleen, kidney, tonsils, and lymph nodes are the best tissues for virus collection. Pig leukocyte cells, bone marrow cultures, porcine alveolar macrophages, and porcine blood monocytes can all be used in ASFV culture. Conventional and real-time PCR have been developed for the detection of ASFV, and multiple primer pairs have been developed to create a rapid diagnostic tool (53–56). A strong IgG response has been detected in domestic swine that survive infection with ASFV (57). As such, serological assays can also be very useful, especially in endemic regions. ELISA, immunoblotting, and indirect fluorescent antibody assays are the most common, and ELISA followed by immunoblotting is often used for international trade purposes.

## Vaccines

It has been particularly challenging to develop an effective ASFV vaccine. To date, no vaccine is available because of a number of key factors, including the lack of identification of protective antigens, incomplete understanding of virus–host cell interactions, and inadequate knowledge relative to the diversity of viral strains currently circulating in natural reservoirs (58, 59). A number of vaccine options have been tried with varying levels of success, including using vaccines with naturally or experimentally deleted genes, subunit vaccines based on recombinant proteins, and DNA vaccines (23). However, none conferred complete protection. A live attenuated vaccine strain was developed and was shown to provide protection against a homologous strain challenge; however, use on the Iberian Peninsula is believed to have been the origin of some low virulence strains that induced a chronic disease form of ASF during the 1960–1995 outbreak (23). Despite this setback, live attenuated vaccines continue to be evaluated for their protective capacity (60, 61).

Knockout ASFV mutants have been evaluated for efficacy although findings have been inconsistent. Afonso et al. (62) describe a highly conserved gene, referred to as *NL*, and found that deletion of the gene from European pathogenic strains resulted in complete attenuation of the virus in domestic swine. *NL*-deleted mutants were created for two highly virulent African strains of ASFV, and inoculation in domestic swine found that these strains retained their virulence, irrespective of the absence of *NL*. These findings suggest that *NL* gene function is not required for these strains of ASFV and that *NL* gene deletion alone is insufficient to engineer live attenuated ASFV vaccines. Gene *9GL* is highly conserved, and *in vitro* evaluation determined that the protein encoded by this gene affects virion maturation and viral growth in macrophage culture (63). The deletion of *9GL* resulted in growth-defective mutants in culture and was found to be highly attenuated in domestic swine. Immunization with a *9GL* knockout virus followed by a challenge with a wild-type ASFV strain resulted in complete protection, and this mutant is being further evaluated as a vaccine candidate for ASFV. The *9GL* gene is also highly conserved and deletion was found to result in complete viral attenuation in swine (64). Vaccination with the mutant strain followed by infection with a wild-type homologous virus resulted in complete protection. Interestingly, however, evaluation of anti-ASFV specific antibodies, ASFV-specific IFNγ response, and circulating cytokine levels found that a complex immune scenario dictates whether infection is established.

Furthermore, A238L is an ASFV immunomodulatory protein that inhibits activation of the NFĸB and NFAT pathways, which are responsible for regulating the synthesis of pro-inflammatory cytokines (65). This protein is believed to be a potent immunosuppressor that may contribute to viral evasion of the host immune response. Unsurprisingly, inoculation of pigs with A238L mutant viruses demonstrated an increase in TNFα, a potent pro-inflammatory cytokine. Much more work is needed to determine whether immunization with viruses with altered immunomodulatory proteins could be harnessed to assist the host immune response against virulent challenge.

Recombinant protein vaccines have also been characterized using a number of relevant viral proteins. p30 and p54 are externally located and involved in virus attachment and virus internalization, respectively (58). Immunization of domestic pigs with either recombinant p54 or p30 proteins induced neutralizing antibodies, but did not protect against lethal challenge and the disease course was unaltered. Combination p54 and p30 vaccines produced both neutralizing antibodies and modified the disease course resulting in a range of protection. Ivanov et al. (66) evaluated 46 peptides that mimic viral proteins for their ability to establish a protective immune response. Vaccination with some combinations of these peptides was found to delay mortality in domestic swine and warrants further investigation. A baculovirus vector expressing the ASFV hemagglutinin was used as a vaccine, and all pigs survived challenge with a virulent virus after immunization (67).

DNA vaccines have also been assessed as an option for ASF, and partial protection was afforded in domestic swine using p54 and p30 as antigens on the construct (68). The robust activation of CD8^+^ cells appears to be extremely important for protection.

Exposure to a non-virulent strain in Portugal (OURT88/3 genotype 1) followed by a virulent strain (OURT88/1 genotype 1) conferred protection against challenge with virulent field isolates from Africa (69). This immunization strategy protected most pigs from both disease development and viremia. The cross-reactivity of the various strains of ASFV can be measured using IFNγ stimulatory assays and provide a strong correlation to the degree of protection conferred.

In addition to evaluating new vaccine preparations, Blome et al. (70) reassessed inactivated ASFV vaccination preparations using modern adjuvants, specifically Polygen and Emulsigen D, which are known to stimulate both humoral and cellular immune responses, including IFNγ. The efficacy of inactivated ASFV vaccines was not improved, and no protection was observed after vaccination followed by challenge with a homologous strain. In fact, vaccinated animals submitted to the disease more quickly, suggesting the possibility of antibody dependent enhancement.

Vaccine development for ASFV is ongoing and challenging due to the range of genetic and antigenic variability as well as the myriad of strategies utilized by the virus to evade the host’s immune response. Further work is essential to develop a vaccine that is both biosafe and provides a high degree of protection across virulent ASFV strains. Subject matter experts believe that live attenuated vaccines are the most promising candidates in the short term due to their experimental successes; however, more studies are required to confirm vaccine safety, capacity to differentiate between naturally infected and vaccinated animals (DIVA), and long-term efficacy (60).

## ASF and the United States

The introduction of ASFV into the United States could negatively affect the domestic swine industry because of morbidity and mortality, the associated losses in production, and restrictions on interstate and international trade. The Foreign Animal Disease Preparedness and Response Plan (FAD PReP), Disease Response Strategy: African Swine Fever put together by USDA APHIS Veterinary Services (71) provides information relevant to all aspects of a disease response in the United States in the event of a viral incursion. The control and eradication strategies are based on four epidemiological principles: (1) prevent contact between ASFV and susceptible animals (primarily *via* quarantine and restricted movement), (2) stop the production of ASFV by infected and/or exposed animals, (3) stop vector transmission, and (4) increase the disease resistance of susceptible animals to ASFV. However, the primary control and eradication strategy are predicated on stamping out (depopulation of clinically affected and in-contact control susceptible swine). Currently, there is no active surveillance being conducted in the United States for ASF. The USDA FAD Prep Document provides information for responders and stakeholders such that they understand the disease agent. Furthermore, a stochastic risk assessment model created by Herrera-Ibata et al. (72) determined the months of highest risk, the origin of the imports of higher risk, and the US states most vulnerable to an ASF introduction. This information can be used to optimize surveillance plans and develop emergency response protocols to help reduce the impact of a potential ASF introduction into the United States.

## Summary of United States Vulnerabilities for the Introduction or Persistence of ASFV

### Risk of Introduction into the United States

Vergne et al. (73) evaluate the pathways for the potential introduction of ASF in China, which maintains over half of the global pig population, and our risk assessment shows similar routes of concern for virus introduction. The legal or illegal movement of live animals or their products, byproducts, or animal feed, or an intentional viral release in an act of bioterrorism comprise the routes of highest concern for ASFV introduction into the United States. It is important to note that to result in an outbreak event, an imported ASFV would need to be released into a susceptible population. An initial outbreak event could occur in domestic or feral swine and then presumably spillover into the other population. Each of these possible routes of introduction is described.

#### Legal Movement of Live Animals

Domestic swine are imported annually from Canada, and Table 1 summarizes the number of animals imported and their purpose. Currently, Canada is ASFV free and, as such, the importation of suids is unlikely to result in an ASFV introduction into the United States.

**Table 1:** Number and purpose of pigs that were imported into the United States from Canada between years 2012 and 2016.

Purpose	Number of animals imported				
	2012	2013	2014	2015	2016
Breeding swine	155,417	196,320	249,214	234,796	150,267
Feeding swine	4,706,866	4,177,805	3,936,987	4,314,664	4,626,477
Direct to slaughter	886,736	824,511	851,002	1,163,884	980,242
Total	5,749,019	5,198,636	5,037,203	5,713,344	5,756,986

#### Legal Movement of Animal Products, Byproducts, and Animal Feed

Animal products and byproducts as well as animal feed that are imported into the United States all require permits upon entry. Products and byproducts that are coming from ASF-endemic regions must be treated in a manner that has previously demonstrated efficacy in destroying ASFV, typically involving heat, pH, or fixation processes. Products and byproducts derived from ASF-free countries can be imported in an unprocessed form. Animal feed from ASF-endemic regions is required to be cooked to a specific temperature and for a specified duration before importation. Products coming from the European Union, which is designated as a low-risk region, can be imported raw if desired; however, documentation is required to certify that the product is coming from an unaffected herd in an unrestricted region.

#### Illegal Movement of Live Animals and Their Products

**Figure 2:**
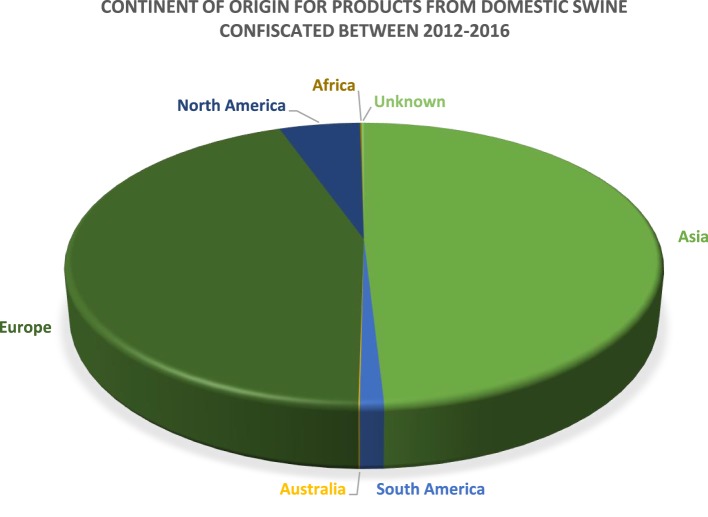
A pie chart depicting the continent of origin for the products confiscated by US Customs and Border Protection between 2012 and 2016 (*n* = 68,594).

The US Department of Homeland Security Customs and Border Protection (CBP) is primarily responsible for the confiscation of illegally imported products and specimens from domestic livestock species. Data provided by CBP depict products and specimens from domestic swine that were confiscated in the cargo or express courier environment, which includes companies such as FedEx and DHL, or *via* international mail facilities, including US postal service. Between calendar years 2012 and 2016, over 68,000 products and specimens derived from domestic swine were confiscated by CBP. The continents of origin for the majority of products and specimens confiscated by CBP are Asia and Europe, which comprise 49 and 44% of the confiscations, respectively. South America, Australia, Africa, and unknown account for ≤1% each, and products and specimens confiscated from North America comprise 5%. These data are summarized in Figure 2.

A large number of products and specimens were derived from continents with regions that are enzootic for ASF. The exact number of products and specimens that are smuggled across the US border is difficult to ascertain, and it can be assumed that the products and/or specimens discovered represent a small subset of the types of goods that are illegally imported into the United States. Due to the types of products confiscated and the regions of the world from which they originate, the illegal importation of domestic livestock products and specimens certainly pose a risk for ASF introduction.

**Figure 3:**
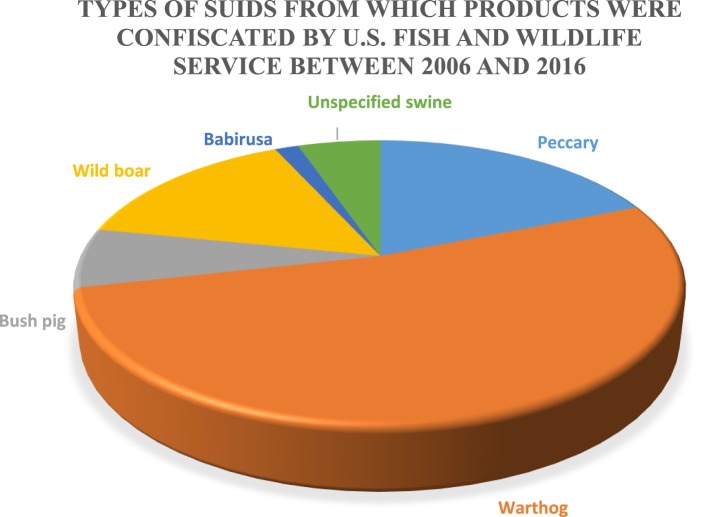
Types of suids from which products were confiscated by the US Fish and Wildlife Services between 2006 and 2016 (*n* = 133).

The US Fish and Wildlife Service (FWS) is responsible for the confiscation of illegally imported wildlife; however, a 1994 report from the Government Accountability Office estimated that 1–3% of illegal wildlife shipments carried by passengers, and 1–10% of illegally imported wildlife in declared cargo shipments are detected (74). This problem is believed to be primarily a result of a limited inspection workforce and budgetary restrictions on overtime, such that ports of entry are often without inspector coverage. FWS provided data relative to wild suid product confiscations in the United States between 2006 and 2016. The types of wild suids from which products were illegally imported and subsequently confiscated by US FWS agents can be found in Figure 3. Warthog products are responsible for more than 60% of confiscations followed by wild boar, bush pigs, unspecified swine products, and babirusa; however, the sample size is small because of the specific nature of the data (wild suids) and because not all illegal imports are likely detected.

**Figure 4:**
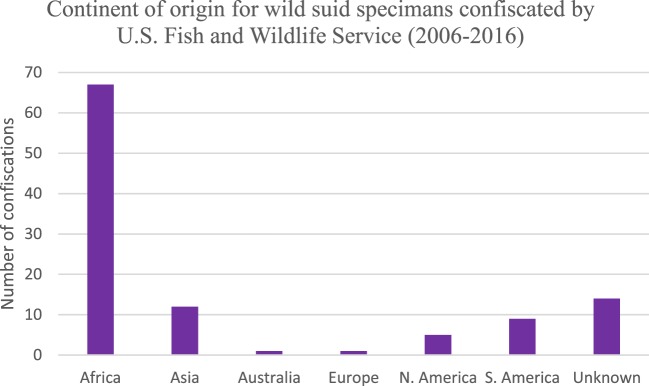
Number of wild suid specimens seized by the US Fish and Wildlife Service between 2006 and 2016 based on continent of origin (*n* = 133).

The majority of products seized by FWS in the United States were those that originated on the continent of Africa (Figure 4). Approximately 25% of confiscations were of products derived from North and South America as well as unknown countries of origin. Asia, Australia, and Europe comprised 13% of confiscations. A large proportion of all confiscated products were derived from continents, which are endemic for ASFV (or unknown); hence, illegal animal/animal product transport presents a risk for ASFV introduction.

#### Bioterrorism

Bioterrorism is described as the intentional release or dissemination of bacteria, viruses, or toxins that cause morbidity or mortality events in humans, other animals, or plants. Due to the tremendous value of the domestic swine industry in the United States, the high morbidity and mortality associated with infection, the ease of viral spread due to the endemic status of many countries globally, the stability of the virus in chilled and frozen products, the safety for the individual(s) involved in the release as the pathogen is not zoonotic, and the crippling economic effects attendant with an introduction, ASFV is a potential candidate to be released in an act of bioterrorism. This route of introduction is difficult to prevent and as such spotlights the need for robust surveillance systems in both domestic and feral swine to ensure rapid detection and differential diagnosis.

### Factors that Complicate Eradication Efforts following Introduction

The risk of ASFV introduction to the United States is low (72). Following a potential introduction, however, ASFV establishment, even short-term establishment, is an open question. ASF has never been found in the United States, but it has successfully taken hold in areas of introduction around the world. Transmission cycles and viral ecology often differ in different locations, demonstrating at least some flexibility for the virus to persist in a range of climates, with or without tick vector involvement, and with or without a wild suid component (75). Climate would not limit ASFV establishment in the United States, and there are tick species that could potentially play a role in viral maintenance (76). The presence of backyard swine and feral swine could also aid in short-term establishment similar to what has been seen elsewhere (43). The biosecure nature of the US commercial swine industry would likely detect and limit ASF transmission without long-term establishment, but economic consequences could still be significant.

Feral swine, which are found in a large number of states, present a risk because of their free-roaming behavior and omnivorous diets and, in the event of a viral incursion, would likely contribute to amplification and transmission events to other feral swine or their domestic counterparts. Soft tick species in the *Ornithodoros* genus that are native to the United States also present an element of complexity as their competence in a field setting remains largely unknown but could substantially complicate viral persistence and disease eradication. These elements are described in detail below.

#### Feral Swine

**Figure 5:**
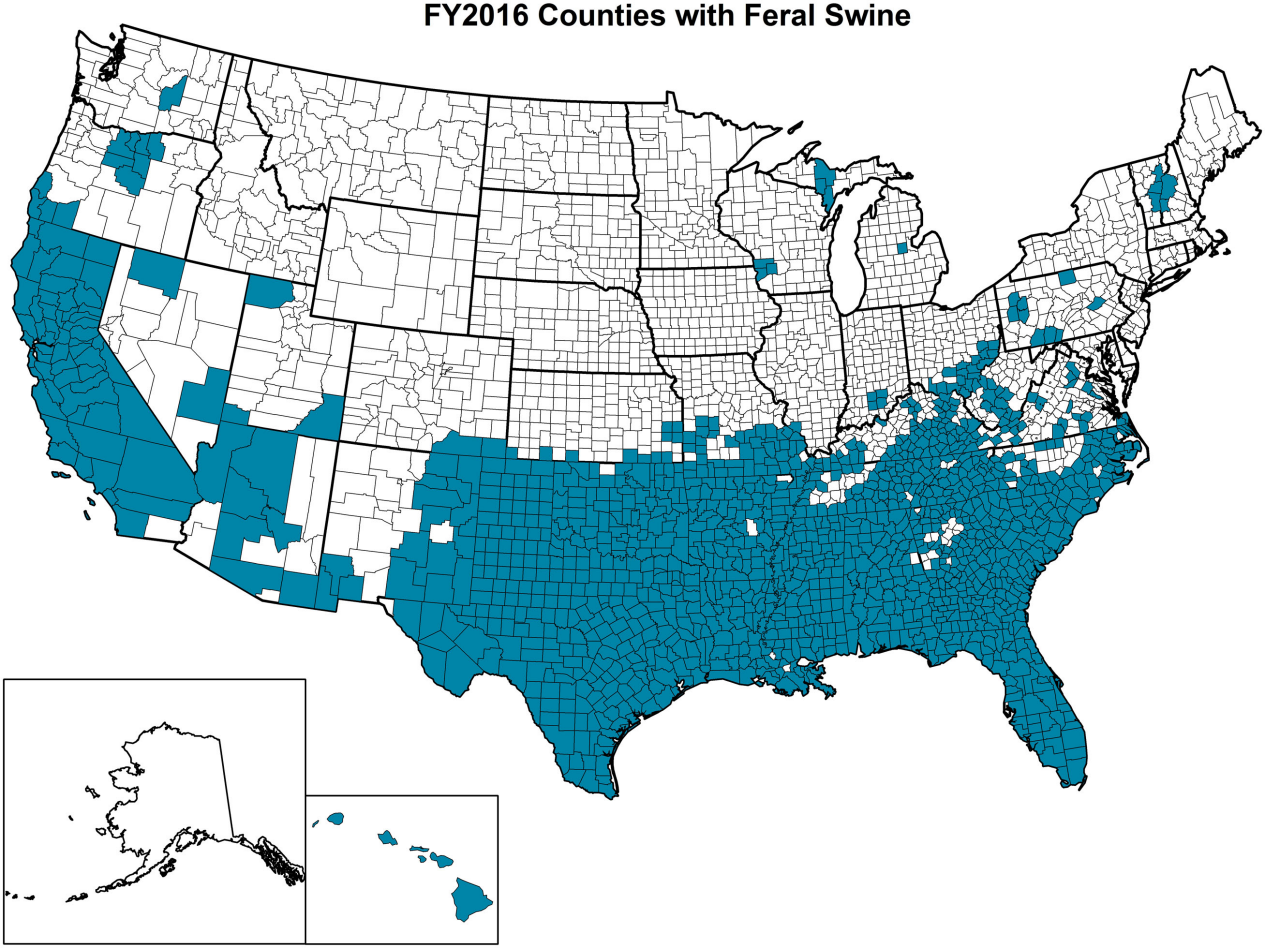
Counties highlighted in blue within each state of the United States where feral swine have been found. (Figure courtesy by APHIS National Feral Swine Damage Management Program.)

The OIE defines feral animals as those that do not live under human supervision or control but have a phenotype that was selected by humans (77). Feral swine (*Sus scrofa*) include released and escaped domestic swine, truly wild Eurasian boars, and their hybrids and are believed to have been originally brought to the United States in the 1400s (78). APHIS experts estimate that over 6 million feral swine roam within at least 35 states in the United States with California, Florida, Oklahoma, and Texas having the largest populations (Figure 5). In addition to being an invasive species, feral swine can damage the environment and agricultural operations; alter ecosystems with their rooting behavior that can be detrimental to threatened and endangered species; and pose a health threat to humans, domestic livestock, wildlife, and companion animals as a result of the type of pathogens that they are capable of carrying and transmitting (79, 80). Studies involving global positioning system (GPS) collared feral swine demonstrated that they contacted domestic swine, and digital images indicated that feral swine attempted to enter pens containing domestic female pigs (52). These types of interactions, which are unsurprising because of the gregarious nature of both domestic pigs and feral swine, increase the risk of pathogen transmission events (81).

Typically male feral swine live a solitary life, while reproductively active females live in small groups with their young, referred to as sounders. Contact rates within and between sounders have been studied using GPS devices, and not surprisingly, contact rates are much higher amongst members of the same sounder compared to those between animals of different sounders (82). However, it has further been shown that sounder home ranges often overlap extensively (83). Sounder interaction is reduced when sounders are separated at distances >2 km, and as such, disease transmission is expected to be reduced between sounders at this distance and nearly negligible between sounders separated by >6 km. Based on these data, the quarantine radius surrounding a positive premise is likely to be at least 2 km, although feral swine activity would be but one factor to consider when determining quarantine size. Certainly, other factors may exist that lead to clustering, such as water availability or baiting activities, among others. Lone boars have been shown to have much larger home ranges compared to sounders and are far more likely to move great distances (84). Furthermore, feral swine densities should also be accounted for as movement may be influenced by density.

Bait stations have been considered as an alternative to fencing for containing feral pigs during culling activities; however, once evaluated empirically, it was found that baiting is not a suitable alternative as only 62% of feral swine trapped within proximity of the bait station used it (85). Baiting can be effectively used to describe patterns of swine movement, facilitate observations, and improve the outcome of removal programs. Interestingly, culling activities did not appear to greatly impact feral swine movements.

In the event of a disease outbreak that affects swine (either exclusively or in conjunction with other livestock species), feral swine could be problematic. Fencing types that can effectively contain feral swine have been evaluated, and hog panels have been found to be highly effective (86). These panels have been found to be effective even when feral swine motivation to escape is increased due to human intervention. In addition, they are relatively quick and cheap to erect—both of which are crucial components in the event of a disease outbreak. While fencing shows promise, it is an option typically reserved for a small, localized scale, such as the area surrounding a single positive farm.

Knowledge derived from ecological and behavioral experiments would be employed, and information specific to the infected premise would be utilized to make an informed decision regarding the frequency and nature of visitation between domestic livestock and feral swine. This information would be used in conjunction with data on other factors such as other nutrient accessibility and feral swine densities (if density data are available) to determine the appropriate spatial scale of fencing or surveillance. Feral swine home ranges can vary dramatically based on the habitat complexity and the availability of food, water, and shelter. For example, a study on feral swine movement in multiple regions of Texas found that the area used by GPS collared individuals could range from 4.5 to 22.23 km^2^ depending on location and season (87). Fencing has been successfully used on large scales to exclude feral swine from a national park in Hawaii [>75 km (88)] and from a national monument in California [42 km (89)], although these were erected over a time frame that was longer than required for a typical outbreak situation. Fencing can also be used to control movement. It is likely that a perimeter fence would be erected around the infected premise with the aim to enclose all feral swine that may have direct or indirect contact with animals from the infected premise before targeted removal of all feral swine within the fenced region. Culling activities would likely be initiated immediately in an attempt to contain disease transmission. Sounders and lone boars that live outside, but near, the quarantine region would likely be closely monitored to evaluate disease transmission and may be subject to prophylactic culling. Outbreak specific characteristics would be important to include, such as the amount of time that has elapsed since the first case, the virulence of the ASFV strain, and the density of both domestic and feral swine, among many other components.

An ecological model developed in Europe showed that conventional wild boar management approaches such as banning feeding and targeted hunting of reproductively active females became slowly effective over multiple generations (90). As such, a buffer of 100–200 km was necessary to compensate for the forward spread of disease until the measures became effective. However, massive population destruction (>80% of the population in the control region within 4 months) or immediate removal of infectious carcasses reduced the buffer zone to <50 km. A hybrid approach of the control methods would result in an intermediate buffer zone width. Of note, hunting as a means to control population and reduce the spread of ASF is very effective, but all efforts should be made to reduce dispersal during this period as the gains made in ASF control *via* population reduction can be quickly offset by wild boar movement and subsequent introduction of ASFV into naive populations (42).

Controlling and/or eradicating disease outbreaks in feral or wild populations is extremely difficult for a number of reasons. Informed decision-making in the absence of knowledge or facts is often required in these types of settings, and as such a systems approach can be used to inform resource allocation and a systematic perspective (91). The publication by Delgado et al. (91) was written with classical swine fever (CSF) in mind; however, many of the components would likely be similar for ASF.

**Figure 6:**
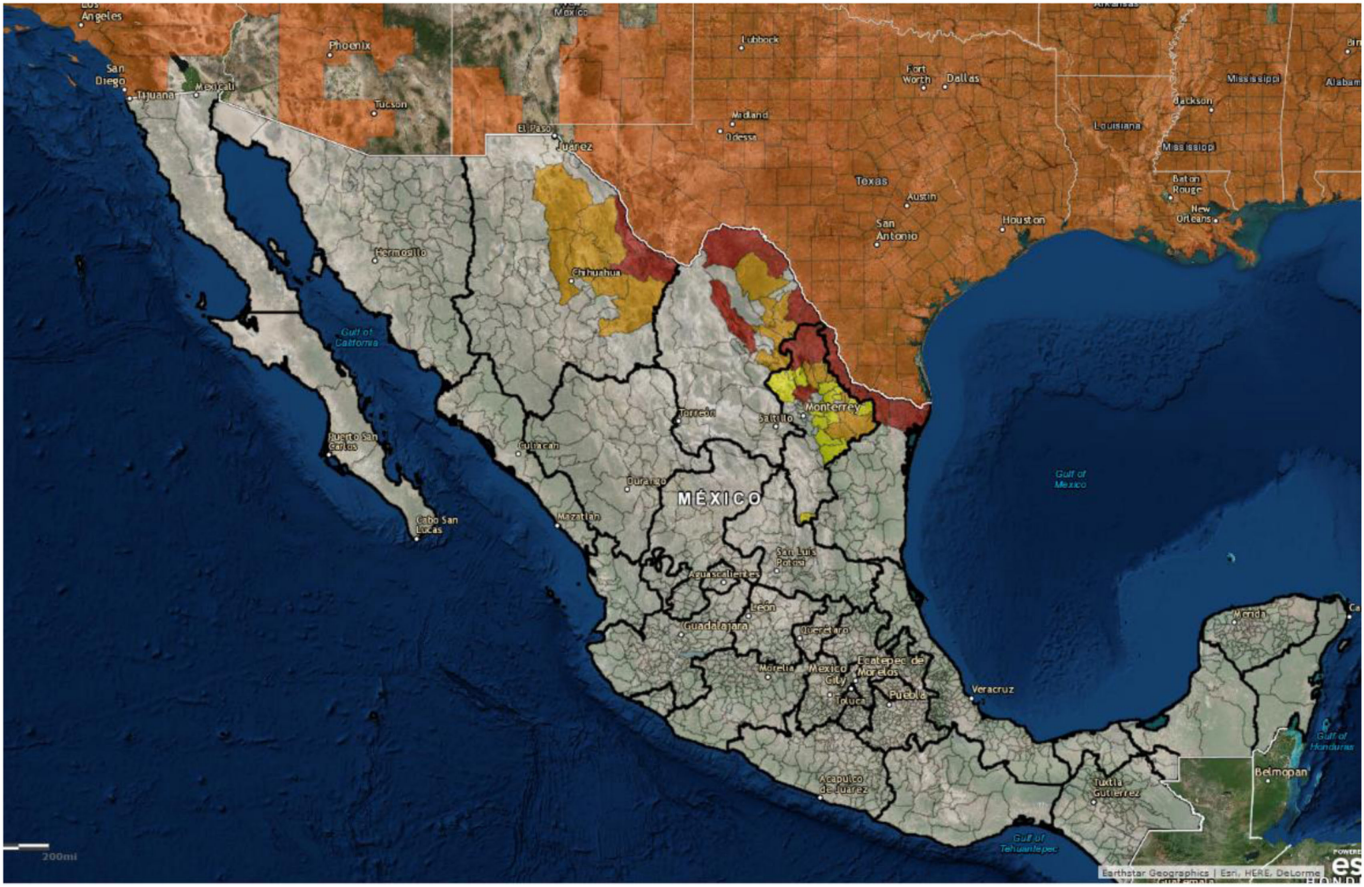
Geographical distribution of feral swine in Mexico (2012): red = high density (>2,000 pigs/county), orange = moderate density (500–2,000 pigs/county), and yellow = low density (<500 pigs/county). (Figure courtesy by APHIS National Feral Swine Damage Management Program.)

The United States is neighbored by two other countries, and feral swine populations move back and forth between countries on both the northern and southern borders. For example, it is not known if a detailed census of feral swine populations throughout Mexico has been done, but there are populations along the United States–Mexico border that are contiguous with the US feral swine population. Figure 6 shows the distribution of feral swine in Mexico based on the subjective reports from the agriculture department of each municipality. Feral swine have been seen moving back and forth across the border along some stretches, depending on the landscape. While both Mexico and Canada are considered ASFV free, it still presents a concern that the borders are porous allowing for movement of feral swine between the countries along both borders. In the event of viral incursion in the United States, Mexico, or Canada that spills over into the feral swine population, this movement will present challenges related to disease control and eradication. Semiquantitative risk assessments have been developed to evaluate the risk of ASF introduction into the EU by wild boar movements as ASF is now considered endemic in much of Eastern Europe (92). In the event of an ASFV introduction in either Canada or Mexico, this type of modeling approach could be used to evaluate the risk of virus introduction into the United States by feral swine.

#### Ornithodoros Ticks in North America

Tick families of veterinary and medical importance include the Ixodidae, which are commonly known as hard ticks, and Argasidae, which are commonly referred to as soft ticks. Several soft tick species in the genus *Ornithodoros* are known vectors of ASFV, and have a nidiculous lifestyle, which indicates their preference to reside in the nest or burrow inhabited by their vertebrate hosts (93, 94). Their lifecycle involves immature and adult male and female stages that take short, repeated blood meals (95). Mated female soft ticks use the blood meal to produce eggs that are laid in a suitable habitat. Adult *Ornithodoros* ticks can live for several years without feeding, and their distribution tends to overlap the geographic range of their hosts (76).

Five species of *Ornithodoros* ticks are found in the United States. *O. coriaceus, Ornithodoros hermsi*, and *O. parkeri* occur in the western and Midwestern regions of the United States and *O. turicata* and *Ornithodoros talaje* are found in the arid regions of the southern United States (10, 96). Laboratory investigations reviewed by Kleiboeker and Scoles (33) demonstrated that *O. coriaceus, O. parkeri*, and *O. turicata* were capable of becoming infected with ASFV and *O. coriaceus* was competent in transmitting the virus to naive domestic swine. Of particular concern is *O. turicata*, found in Arizona, California, Colorado, Florida, Kansas, New Mexico, Oklahoma, Texas, and Utah. These states also provide suitable habitat for large numbers of feral swine, which presents an opportunity for the maintenance of ASFV by *O. turicata* in the event of a viral incursion with the involvement of feral swine (97).

Because of their short-feeding duration and nidiculous lifestyle, the global distributions of soft ticks can be challenging to determine; however, their capacity to transmit pathogens makes this information of the utmost importance. A regional model using spatial multicriteria decision analysis to identify geographical areas that are suitable for specific species of *Ornithodoros spp*. was created by Vial et al. (98). This model was developed for the Western Palearctic region; although in the event of ASFV introduction into the United States, this methodology could be applied to native *Ornithodoros* ticks to determine species and regions of concern.

In addition to the competent *Ornithodoros* species found in the United States, *O. puertoricensis* is found in the Caribbean, specifically Jamaica, Haiti, the Dominican Republic, and in Panama (99) and can be infected with, and transmit, ASFV to susceptible domestic swine (33). The porous border between the United States and Mexico provides further complexity in the event of an introduction of ASFV in either country, and soft tick vectors could play a role as an epidemiological bridge as they might be transported to disease-free regions. It is important to note that although soft ticks engorge rapidly and tend to drop off their host after completion of the blood meal, reports of host infestation as “stowaways,” including feral swine, captured outside of their nest or burrow has occurred (100, 101). Thus, this potential route of viral introduction or spread is worth mentioning. Furthermore, they are often promiscuous in their host preferences and have occasionally been recovered from birds, which also pose a risk for disease translocation (10).

## Concluding Remarks

African swine fever virus introduction (either accidental or purposeful) to the United States could cause severe morbidity and mortality in domestic swine. Furthermore, the trade implications associated with ASFV in domestic swine are substantial and could severely affect the pork industry. The current regulatory systems in place for the importation of live animals, animal products, byproducts, and feed are comprehensive, involving considerable Federal oversight and encompassing information relevant to the country of origin, the product to be imported, and the species involved must conform to research methods that effectively demonstrate the deactivation of ASFV. Despite the robust regulatory framework, the illegal importation of animals and their products is in its very nature difficult to control, manage, or regulate. Semiquantitative approaches can be used to evaluate the risk of disease introduction *via* the illegal importation of pork and pork products, and modeling in the European Union suggests that this channel certainly serves as a risk for ASFV importation and subsequent introduction (102). Bioterrorism is another potential route of introduction. Given the complexities of preventing accidental or purposeful ASFV introduction into the United States, vigilant observance of domestic livestock and rapid reporting and differential diagnosis are necessary in the event of a disease detection in pigs. Channels for rapid communication and diagnostics already exist through state and national veterinarians and laboratories as evidenced by a pilot study in which samples were collected from culled feral swine and evaluated for the presence of ASFV. The evaluation of samples for ASFV suggests that labs are proficient in diagnostic techniques necessary for viral detection.

African swine fever virus introduction, or spillover, into feral swine populations would heavily complicate eradication. Furthermore, unrestricted movement of feral swine across porous borders presents a challenge in the event of an ASFV incursion into any countries that share borders. The presence of competent biological vectors, *Ornithodoros* ticks, further complicates the control and eradication of ASFV upon introduction to a new region. These ticks are often long lived and are believed to play an important role in viral maintenance and may contribute to the development of endemicity in a specific region. *O. coriaceus, O. parkeri*, and *O. turicata* are present in the United States and have been found to be capable of ASFV infection and in the case of *O. coriaceus*, ticks are capable of transmitting the virus to susceptible swine (33, 96). *Ornithodoros dugesi* are found in Texas and northern Mexico, and *O. talaje* are found in the southern United States; however, neither species has been evaluated for its competence as a biological vector for ASFV. Further laboratory studies should be designed to evaluate the ability for *O. dugesi* and *O. talaje* to become infected with various strains of ASFV and to characterize the ability for *O. parkeri* and *O. turicata* as well as *O. dugesi* and *O. talaje*, pending their capacity to become infected, to transmit infection to susceptible domestic swine. In addition, expanded analyses to explore the distribution of competent *Ornithodoros* ticks in relation to dense commercial pig production regions as well as high populations of feral swine are needed. Moreover, determining host preferences for competent vector species is important to characterize risk. The lack of a vaccine for ASFV makes disease control and eradication substantially more difficult, and as such, efficacious vaccine development is a high priority. Characterization of antigenic viral proteins that contribute to a host immune response and a determination of immune mechanisms that lead to protection are extremely important and foundational for the quest of a vaccine.

Currently, the United States does not have any active surveillance protocols for ASF in domestic or feral swine. The risk of introduction is believed to be low because the disease is not currently present in the western hemisphere. In addition, the introduction of ASFV into a naive population is typically accompanied by severe mortality such that passive surveillance, sampling of dead pigs, would likely be sufficient to detect an ASFV incursion (103, 104). Importantly, during calendar year 2017, the Foreign Animal Disease Diagnostic Laboratory (FADDL) at Plum Island Animal Disease Center performed only two cases requesting ASF testing and both were negative (Personal communication). Of note, however, a stochastic model used to evaluate transmission of ASFV within a population found that the virus may be circulating in a herd for several weeks before a marked increase in mortality is observed, which limits the usefulness of mortality data as a means of early detection in an outbreak scenario (105). It may also be useful to compare the conditions in the United States to those in Europe to determine whether the buffer zones necessary to quell an ASF outbreak in Europe (90) would be similar to those required for an outbreak in the United States.

USDA Veterinary Services have outlined a surveillance program for CSFV in domestic swine that could be harnessed to evaluate ASFV in the event the risk of introduction increases. The objectives are as follows: (1) surveillance for rapid detection of CSFV in US swine, (2) monitor the risk of introduction of CSF into US swine, (3) surveillance of international CSF status, and (4) surveillance to document freedom of CSF (71). Unthrifty pigs, considered to be those that gain weight poorly or are otherwise somewhat sickly, are often sold to off-market vendors. APHIS Veterinary Services field staff or other cooperating personnel collect tonsil samples in these markets as a way to survey for infectious agents, including CSFV. This method is deemed to be an effective surveillance strategy as poor-doing pigs from surrounding regions are often consolidated in these markets, which makes for an efficient means of sampling sickly pigs from a wider geographical area. Furthermore, high-risk areas, designated by APHIS as regions with garbage feeding operations, backyard swine operations, feral swine hunting clubs, military bases, international air or sea ports, farming operations utilizing an international labor force, and/or corporations engaging in international swine movement, are subject to active surveillance protocols *via* tonsil collection; 25 states are considered high risk. All garbage feeder operations in the United States are licensed and regularly inspected, and heat treatment of all feed is mandatory. Texas and Florida are considered particularly high risk, and as such, two swine slaughter establishments in Florida and three in Texas randomly collect blood, which is sent to the FADDL for further testing, especially from pigs in the southern portion of each state, light-weight pigs, or those in transition. This active surveillance for CSFV in domestic swine could readily be extended to include surveillance for ASFV as samples are already being collected and transported to FADDL for screening purposes.

Moreover, feral swine are also surveyed as a preventative and early sentinel in the event of a CSFV intrusion. For fiscal year 2017, USDA APHIS National Feral Swine Damage Management Program is rolling out a targeted surveillance plan in which existing feral swine populations, domestic hog production areas, and landfills are used as criteria for determining priority of feral swine samples collected for disease surveillance. Counties are weighted based on the presence or absence of each of the aforementioned criteria. This type of targeted surveillance is crucial to allow for the efficient use of time and resources and to increase the probability of detecting an outbreak early (106, 107). Samples are collected *via* culling operations as well as from hunter-killed pigs, and serology is performed to evaluate the presence of CSFV antibodies. Again, expanding this program to include ASFV screening in feral swine may be beneficial, especially if the perceived risk of ASFV entering the United States increases, as it would likely contribute to early detection in the event of a viral incursion and would be far less costly than an ASFV-exclusive active surveillance protocol.

Importantly, several strains of *Ornithodoros* soft ticks are found in regions with high feral swine populations, especially Texas, Florida, and Oklahoma (*O. turicata*) and California (*O. coriaceus*). Both of these tick species have been shown to become infected with ASFV and *O. coriaceus* is capable of transmitting the virus to susceptible domestic swine >500 days after infection (33). *Ornithodoros* ticks are permissive to ASFV infection with varying capacities for infection; thus, it is hypothesized that other *Ornithodoros* species ticks found in the United States are competent ASFV vectors. The high density of feral and domestic swine in these regions and a strong likelihood for overlapping distribution with potential soft tick vectors further the notion that an active surveillance protocol may be useful and contribute to early detection in the event that ASFV emerges in the United States.

It is important to note that much of the spread of ASF through Eastern Europe and the Caucuses region is likely driven by anthropogenic factors, such as the movement of infected pigs and their products as well as *via* swill feeding (108). However, ticks cannot be overlooked as they are believed to have maintained ASFV in Portugal over a 6-year period during which time the country was declared ASF free prior to the re-emergence of the disease on a single farm (48). The role of vectors in pathogen maintenance and transmission events is often poorly understood, and these long-lived ticks may play a crucial role in conjunction with human activities which likely facilitate ASFV spread.

Sampling of illegally imported and subsequently confiscated, suids, and their products would also provide meaningful data relevant to the types of pathogens being imported. The General Accounting Office (74) estimated that 1–3% of illegally imported wildlife carried by passengers was detected and 1–10% of illegally imported wildlife in declared cargo shipments. Smith et al. (109) performed a pilot study evaluating zoonotic agents in confiscated animal products from John F. Kennedy airport in New York, New York, and found that multiple strains of retroviruses and herpesviruses were present in several non-human primate specimens. Knowledge relative to the types of pathogens entering the United States in illegally imported swine products would be useful in understanding risk of both swine-specific pathogens and zoonotic organisms.

African swine fever presents a substantial threat to both domestic and wild suid species. The concern of viral introduction in the United States has contributed to the implementation of a series of preventive measures designed for importation of live animals and their products. Despite extensive research, knowledge gaps exist, and they have been highlighted as areas for future evaluation.

## Author’s Note

Oak Ridge Institute for Science and Education (ORISE) supported by the U.S. Department of Homeland Security (DHS) Science and Technology Directorate (S&T) Homeland Security Advanced Research Projects Agency (HSARPA), Chemical and Biological Defense Division (CBD) under Agreement # HSHQPM-13-X-00174).

## Author Contributions

VB was involved in the development of the idea, collection of the data, data analysis and interpretation, and preparation of the document. SB was involved in idea development and document preparation.

## Conflict of Interest Statement

The authors declare that the research was conducted in the absence of any commercial or financial relationships that could be construed as a potential conflict of interest.
